# A 27-year-old woman presenting with fever and cervical lymphadenopathy

**DOI:** 10.12701/jyms.2026.43.33

**Published:** 2026-05-12

**Authors:** Chae Eun Lee, Hyun-Je Kim

**Affiliations:** 1 Yeungnam University College of Medicine, Daegu, Korea; 2 Division of Rheumatology, Department of Internal Medicine, Yeungnam University College of Medicine, Daegu, Korea

## Case presentation

A 27-year-old woman presented with a 2-week history of intermittent fever. She also complained of neck mass sensation. Although palpable masses on both sides of the neck were noted on physical examination, she described a subjective feeling of fullness deep within the neck and reported discomfort when shaking her head. She had no significant medical history and was not taking any medication prior to presentation. Before referral to the rheumatology department of the hospital, laboratory tests performed at a local medical clinic revealed elevated levels of inflammatory markers, including erythrocyte sedimentation rate (ESR) and C-reactive protein (CRP), and the patient was referred for further evaluation. During hospitalization, the patient experienced recurrent high-grade fever up to 39.8°C. She also exhibited a symmetric rash over the cheeks lateral to the nasolabial folds and complained of general pain and arthralgia involving the small joints of both fingers. On examination of the neck, multiple tender, round nodular masses were palpated along the medial aspect of both sternocleidomastoid muscles, consistent with cervical lymphadenopathy. Given the persistent fever accompanied by systemic symptoms and lymphadenopathy, the patient was admitted for the evaluation of a fever of unknown origin. The initial diagnostic considerations included infectious diseases, autoimmune or inflammatory disorders, and malignancies such as lymphoma. Comprehensive laboratory tests were performed to evaluate infectious etiologies. In addition, positron emission tomography-computed tomography (PET-CT) was performed to assess the extent of lymphadenopathy and to exclude underlying malignancy or systemic inflammatory disease.

## Differential diagnosis

In patients presenting with fever and generalized lymphadenopathy involving the cervical, thoracic, and abdominopelvic regions, several etiologies should be considered in the differential diagnosis.

### 1. Chronic infectious lymphadenitis: tuberculous lymphadenitis, toxoplasmosis, and Epstein-Barr virus reactivation

In patients presenting with persistent fever and generalized lymphadenopathy lasting longer than 1 week, chronic infectious etiologies must be considered in the differential diagnosis. In cases of acute lymphadenitis, empirical antibiotic therapy usually resolves fever and improves lymph node enlargement. However, when fever and lymphadenopathy persist despite initial management, additional etiologies should be considered. The infectious causes of generalized lymphadenopathy include viral, bacterial, mycobacterial, and parasitic infections. Viral etiologies include human immunodeficiency virus (HIV), Epstein–Barr virus (EBV), and cytomegalovirus (CMV). Bacterial causes include *Salmonella*, *Streptococcus*, and *Staphylococcus* species, as well as syphilis. Mycobacterial infections, including *Mycobacterium tuberculosis* and nontuberculous mycobacteria, should also be considered. In addition, *Toxoplasma gondii* is an important cause of parasitic infections. In the present case, laboratory investigations were performed to evaluate possible infectious etiologies. The patient underwent blood tests, including complete blood count, ESR, CRP, EBV, CMV, HIV, venereal disease research laboratory test, *Toxoplasma* serology, hepatitis viral marker, and interferon-gamma release assays. Although inflammatory markers were elevated, all serological tests for infectious diseases were negative (Table 1), making an infectious etiology less likely.

### 2. Lymphoproliferative disease: malignancies including lymphoma, squamous cell carcinomas of the head and neck, and thyroid cancer

**Fig. 1.**
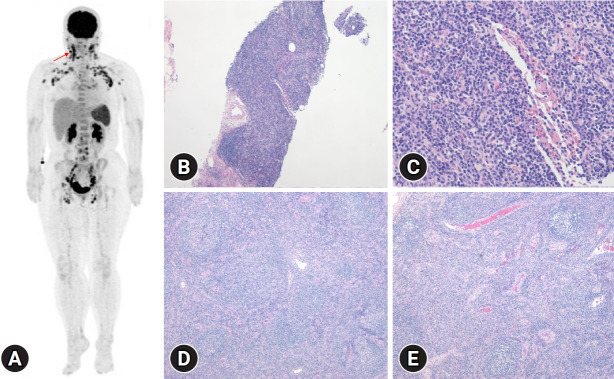
(A) Multiplanar reformation of the positron emission tomography-computed tomography scan showing intense fluorodeoxyglucose-avid uptake in the right level II cervical lymph node (arrow). (B) Core needle biopsy (CNB) specimen exhibiting fragmented lymphoid tissue with preserved follicular architecture (hematoxylin and eosin [H&E] stain, ×40). (C) High-power view of the CNB specimen demonstrating moderate plasma cell infiltration in the interfollicular regions, suggesting an autoimmune-related process (H&E stain, ×200). (D) Excision biopsy showing marked intrafollicular hyperplasia with numerous enlarged, reactive germinal centers (H&E stain, ×40). (E) Excision biopsy highlighting significant interfollicular hyperplasia and paracortical expansion. Note the prominent vascular proliferation and dense polymorphous lymphoid cell infiltration, characteristic of architectural distortions encountered in systemic lupus erythematosus (SLE)-associated lymphadenopathy (H&E stain, ×40).

In patients with generalized lymphadenopathy, it is essential to exclude malignancies, particularly lymphoproliferative disorders. Malignant causes include lymphomas (both Hodgkin and non-Hodgkin lymphoma) and solid tumors involving the head and neck region, including thyroid malignancy, which may present as a neck mass. Imaging studies play a crucial role in evaluating these patients. Computed tomography of the neck, chest, and abdomen can be performed to assess the extent of lymphadenopathy. Alternatively, as in the present case, PET-CT may be useful to evaluate the systemic distribution of the disease and identify the most appropriate site for biopsy. In this patient, PET-CT demonstrated numerous intensely fluorodeoxyglucose (FDG)-avid lymph nodes of varying sizes involving the bilateral cervical, periauricular, supraclavicular, axillary, retroperitoneal (abdominopelvic), and bilateral inguinal regions, indicating generalized lymphadenopathy (Fig. 1A). These findings led to the differential diagnoses of inflammatory or infectious lymphadenopathy (e.g., Kikuchi disease) and lymphoproliferative disorders, including lymphoma involving multiple nodal regions on both sides of the diaphragm.

Core needle biopsy (CNB) of an axillary lymph node was initially performed to establish a definitive diagnosis. Histopathological examination revealed an atypical lymphoplasmacytic proliferation. In addition, the paracortical area showed mild cytologic atypia of T lymphocytes with prominent vascular proliferation, raising concerns regarding angioimmunoblastic T-cell lymphoma (AITL) (Fig. 1B, 1C). Therefore, an excisional biopsy was recommended for further evaluation. Subsequently, an excisional biopsy of a right level II cervical lymph node was performed. Histological examination revealed follicular hyperplasia with moderate plasma cell infiltration in the interfollicular areas, consistent with autoimmune-related changes. In addition, the sections showed paracortical expansion of polymorphous lymphoid cell infiltration with prominent vascular proliferation, along with plasma cell infiltration in both the interfollicular and intrafollicular regions. Some follicles exhibited regressed germinal centers with sclerotic vessels, which are features reminiscent of Castleman disease (CD) (Fig. 1D, 1E).

### 3. Lymphoproliferative disease: autoimmune lymphoproliferative disease including idiopathic multicentric Castleman disease, immunoglobulin G4-related disease, and sarcoidosis

Once malignancy has been reasonably excluded in patients with generalized lymphadenopathy, autoimmune and immune-mediated disorders primarily involving lymph nodes should be considered in the differential diagnosis.

CD is a rare lymphoproliferative disorder first described by Castleman et al. in 1956 [1]. It is classified as unicentric CD and multicentric CD (MCD), the latter of which includes human herpesvirus-8-associated MCD and idiopathic MCD (iMCD). Histopathologically, CD is categorized into the hyaline vascular, plasma cell, and mixed variants. MCD is a systemic disorder characterized by polyclonal plasmacytic proliferation and dysregulated immune activation, often presenting with fever, generalized lymphadenopathy, splenomegaly, edema, weight loss, and multiorgan dysfunction in severe cases [2].

Immunoglobulin G4-related disease (IgG4-RD) is another important differential diagnosis characterized by tumor-like swelling of affected organs, often involving the exocrine glands and extranodal tissues, along with elevated serum IgG4 levels [3]. Lymph node involvement is also common. Pathologically, patients with IgG4-RD present with dense lymphoplasmacytic infiltration, irregular fibrosis, obliterative phlebitis, and infiltration of IgG4-positive plasma cells. The diagnosis of CD requires the exclusion of other diseases with overlapping histological features, including lymphoma, POEMS (polyneuropathy, organomegaly, endocrinopathy, monoclonal protein, and skin changes) syndrome, primary lymph node plasmacytoma, follicular dendritic cell sarcoma, systemic lupus erythematosus (SLE), Sjögren syndrome, and IgG4-RD. Sarcoidosis should be considered as a differential diagnosis. It is a multisystem granulomatous disease that most commonly involves the lungs but may also affect the lymph nodes, skin, and eyes [4]. Histologically, it is characterized by non-caseating granulomas, and its clinical manifestations, including cough, dyspnea, ocular symptoms, arthritis, sensory changes, and skin lesions, vary depending on the organs involved [5].

In the present case, based on the clinical presentation and histopathological findings, the diagnosis was considered inconsistent with iMCD, IgG4-RD, and sarcoidosis, thereby narrowing the differential diagnosis.

### 4. Connective tissue disease-related secondary lymphoproliferative disease, such as systemic lupus erythematosus

Given that extensive evaluations were performed to exclude alternative etiologies, including infection and malignancy, the patient’s clinical and laboratory findings were considered compatible with an autoimmune-mediated disease process. The patient presented with fever, generalized lymphadenopathy, and erythematous skin lesions involving the cheeks and dorsal aspects of the hands. Laboratory investigations included rheumatoid factor (RF), anti-cyclic citrullinated peptide antibody (ACPA), antinuclear antibody (ANA), anti-double-stranded deoxyribonucleic acid antibody (anti-dsDNA Ab), anti-Smith Ab, anti-histone Ab, anti-phospholipid Abs (including anti-cardiolipin), complement levels (C3 and C4), and immunoglobulin levels (immunoglobulin G [IgG], immunoglobulin A [IgA], immunoglobulin M [IgM], and IgG4). The laboratory results were as follows: white blood cell count, 2,350/µL; hemoglobin, 11.6 g/dL; platelet count, 206,000/µL; ESR, 42 mm/hour; fluorescent ANA titer, 1:1,280; RF, 2.4 IU/mL; ACPA, <8 U/mL; C3c, 19.5 mg/dL; C4, 3.9 mg/dL; IgG, 2,222 mg/dL; IgA, 353 mg/dL; IgM, 155 mg/dL; IgG4, 8.8 mg/dL, anti-dsDNA IgG Ab, 39.4 IU/mL; anti-Smith Ab, 200 U/mL; and anti-cardiolipin IgG Ab, 17.2 IgG phospholipid units/mL. Major laboratory findings are summarized in Table 1. A skin biopsy of dorsal hand lesions revealed interface dermatitis, a histopathological finding commonly associated with autoimmune connective tissue diseases. Despite the presence of arthralgia, no objective evidence of arthritis was identified in the wrists, fingers, or ankles, and serological markers associated with rheumatoid arthritis were negative, making rheumatoid arthritis unlikely. In addition, the patient did not report any clinical features suggestive of Sjögren syndrome, such as xerostomia, xerophthalmia, or salivary gland enlargement, which further reduced the likelihood of this diagnosis.

Taken together, the patient’s clinical presentation, serological profile, and histopathological findings supported an autoimmune etiology, whereas other connective tissue diseases were considered less likely based on the absence of defining clinical or laboratory features.

## Diagnosis

Based on the clinical presentation, lymph node histopathological findings, and serological results described above, the patient was diagnosed with SLE according to the 2019 European League Against Rheumatism/American College of Rheumatology (EULAR/ACR) classification criteria [6]. In patients with fever and generalized lymphadenopathy, it is essential to systematically evaluate chronic infectious lymphadenitis and primary lymphoproliferative disorders, including both malignancy- and autoimmune-related conditions. Once these conditions are reasonably excluded, the possibility of a secondary lymphoproliferative process associated with a systemic autoimmune disease should be considered. In the present case, the patient accrued 23 points on the 2019 EULAR/ACR classification criteria: fever (2), skin rash consistent with acute cutaneous lupus manifested by malar rash and cutaneous hand lesions (6), leukopenia (3), low C3 and C4 levels (4), anti-phospholipid Ab positivity represented by positive anti-β2 glycoprotein I IgG results (2), and SLE-specific Ab positivity represented by anti-dsDNA Ab or anti-Smith Ab positivity (6). This total exceeds the classification threshold of 10. A positive ANA test served as the entry criterion (Table 2). These findings, in conjunction with the overall clinical and pathological features, were considered to have a significant diagnostic value.

## Treatment and prognosis

The patient was treated with high-dose corticosteroid therapy, namely intravenous methylprednisolone 1 mg/kg (60 mg/day). Following treatment initiation, the patient’s fever subsided, and the neck mass associated with the lymphadenopathy showed significant improvement. To maintain disease control while tapering the corticosteroid dose, weekly methotrexate and azathioprine were administered as steroid-sparing immunosuppressive agents. The patient is currently receiving methotrexate 10 mg weekly and azathioprine 100 mg daily, with gradual tapering of the corticosteroids to prednisolone 10 mg once daily. With this treatment regimen, the patient’s fever and lymphadenopathy-related masses in the cervical and axillary regions markedly improved. The patient continues to receive regular outpatient follow-ups and management.

## Discussion

SLE is a chronic, multisystem autoimmune disease characterized by autoantibody production and immune complex-mediated tissue injury. The term “lupus,” meaning “wolf,” originated from the characteristic malar rash resembling a wolf bite [7]. However, the clinical spectrum of SLE is highly heterogeneous, and constitutional symptoms such as fever and lymphadenopathy may precede or predominate over classic cutaneous manifestations, posing a diagnostic challenge in early stages of the disease. Although malar rash is a hallmark feature of SLE, patients may initially present with nonspecific systemic symptoms, including fever, arthralgia, and generalized lymphadenopathy [8]. In such cases, SLE can closely mimic infectious diseases or lymphoproliferative disorders, requiring extensive diagnostic evaluations. In particular, fever with generalized lymphadenopathy warrants careful assessment to exclude chronic infections, Kikuchi disease, and lymphoproliferative malignancies. Among the autoimmune and inflammatory conditions, iMCD and IgG4-RD are important differential diagnoses. iMCD is characterized by systemic inflammation, polyclonal plasmacytosis, and cytokine dysregulation, and is often accompanied by generalized lymphadenopathy and constitutional symptoms. IgG4-RD typically presents with a tumor-like swelling of the affected organs, elevated serum IgG4 levels, and characteristic histopathological findings, including lymphoplasmacytic infiltration and fibrosis. Differentiating these conditions from SLE is crucial because, despite their overlapping features, they require distinct therapeutic strategies.

This case highlights the diagnostic difficulty of SLE presenting with prominent fever and generalized lymphadenopathy, which can closely mimic malignant lymphoma, both clinically and pathologically. In the present case, the diagnostic impression was further complicated by the initial CNB, suggesting AITL, which substantially complicated the diagnostic process. Nevertheless, the overall clinicopathological and serological profiles supported the diagnosis of active SLE rather than lymphoma or other inflammatory lymphadenopathies. In the present case, the patient fulfilled the 2019 EULAR/ACR classification criteria with a total score of 23 points: fever (2), acute cutaneous lupus (6), leukopenia (3), low C3 and C4 levels (4), anti-phospholipid Ab positivity represented by positive anti-β2 glycoprotein I IgG (2), and anti-dsDNA or anti-Smith Ab positivity (6). This exceeded the classification threshold of 10, with a positive ANA serving as the entry criterion (Table 2).

The most important teaching point in this case was the potential pitfalls of using CNB in the evaluation of autoimmune lymphadenopathy. Although CNB is less invasive and may be useful as an initial diagnostic tool, it may be misleading when the differential diagnosis includes SLE-associated lymphadenopathy and lymphoma. Small-volume tissue sampling may overrepresent focal atypical lymphoid proliferation, while failing to preserve the overall nodal architecture, which is critical for distinguishing reactive immune activation from true lymphoid malignancies. In the present case, CNB raised concerns regarding AITL because of the atypical lymphoplasmacytic proliferation, paracortical atypia, and vascular proliferation. However, an excisional biopsy revealed follicular hyperplasia, interfollicular plasma cell infiltration, and paracortical expansion with prominent vascular proliferation in an architectural context that was more compatible with autoimmune-related lymphadenopathy. This discrepancy underscores that excisional biopsy is often mandatory when the clinical suspicion of SLE remains high, despite atypical or lymphoma-like findings on small-sample pathology.

Another important aspect of this case is the significance of serological abnormalities in explaining the lymphoma-mimicking presentation. The decreased C3 and C4 levels and anti-dsDNA Ab positivity strongly support active immune complex-mediated systemic inflammation and complement consumption in SLE. These findings are not merely ancillary laboratory abnormalities; they also provide a pathophysiological context for fever, cytopenia, and lymphadenopathy. In this setting, the serological profile favored active SLE over other causes of necrotizing or reactive lymphadenitis and was particularly useful in differentiating SLE-associated lymphadenopathy from entities such as Kikuchi disease, in which complement consumption is generally not a defining feature. This integrative approach is particularly important because an isolated pathological interpretation of a limited specimen may not adequately reflect the systemic autoimmune process.

This case emphasizes that SLE should remain in the differential diagnosis of patients presenting with fever and generalized lymphadenopathy, even when the initial imaging and limited biopsy findings raise concerns regarding lymphoma. An accurate diagnosis requires careful integration of clinical manifestations, serological markers, imaging studies, and adequate tissue pathology. When CNB findings are discordant with the overall clinical impression, prompt excisional biopsy may be necessary to avoid misdiagnosis and unnecessary treatment of lymphoproliferative disease. Therefore, this case provides an instructive example of how active SLE can masquerade as a lymphoproliferative disorder and highlights the value of multidisciplinary diagnostic assessment.

## Educational pearls

1. A fever of unknown origin with generalized lymphadenopathy requires a broad differential diagnosis, including chronic infections, primary lymphoproliferative disorders, and autoimmune disease-associated secondary lymphadenopathy.

2. PET-CT is valuable for assessing the extent of systemic lymphadenopathy. However, its cost-effectiveness should be considered. CNB may have limitations in the evaluation of autoimmune lymphadenopathy because inadequate preservation of nodal architecture may lead to misleading interpretations; in such cases, excisional biopsy may be required for definitive diagnosis.

3. Patients with SLE may initially present with fever and lymphadenopathy. Because the classification criteria require the exclusion of alternative diagnoses, careful and systematic evaluation is essential before confirming the diagnosis.

## Question 1

A 27-year-old woman presents to the outpatient clinic with a 2-week history of fever (up to 39°C) and bilateral neck swelling. She also reports fatigue and joint pain in her hands and feet. Physical examination reveals multiple tender cervical lymph nodes along both sternocleidomastoid muscles. A symmetric erythematous rash is noted over both cheeks, sparing the nasolabial folds. Laboratory findings show an elevated ESR and CRP level. The initial infectious workup, including EBV, CMV, HIV, and tuberculosis testing, is negative. PET-CT reveals generalized FDG-avid lymphadenopathy. An excisional lymph node biopsy reveals reactive lymphoid hyperplasia with polymorphous lymphoid infiltration and vascular proliferation without evidence of malignancy.Which of the following is the most appropriate diagnosis?

- A. Angioimmunoblastic T-cell lymphoma (AITL)
- B. Systemic lupus erythematosus (SLE)
- C. Kikuchi disease
- D. Immunoglobulin G4-related disease (IgG4-RD)
- E. Sarcoidosis

B

## Question 2

Physical examination reveals cervical and axillary lymphadenopathy, and PET-CT shows widespread FDG-avid lymph nodes. The initial CNB is inconclusive, and an excisional biopsy is performed. You suspect a systemic autoimmune disease such as SLE.Which of the following findings would most strongly support this diagnosis?

- A. Noncaseating granulomas on lymph node biopsy
- B. Caseating granulomas with central necrosis
- C. Interface dermatitis on skin biopsy
- D. Monoclonal lymphoid proliferation on immunohistochemistry
- E. Storiform fibrosis with IgG4-positive plasma cells

C

## Figures and Tables

**Table 1.** Laboratory findings of the case

Test item	Test result	Unit	Reference range
WBC	2.35	k/µL	4.0–10.0
Hemoglobin	11.6	g/dL	12–16.5
Platelet count	206	k/µL	140–440
ESR	42	mm/hr	0–20
CRP	0.07	mg/dL	0–0.5
AST	74	U/L	10–35
ALT	76	U/L	10–40
BUN	9.2	mg/dL	8–23
Creatinine	0.5	mg/dL	0.7–1.2
LDH	375	U/L	0–250
Albumin	3.71	g/dL	3.5–5.0
A/G ratio	1.2		1–2.1
IgG	2,222	mg/dL	700–1,600
IgA	353	mg/dL	70–400
IgM	155	mg/dL	40–230
IgG4	8.8	mg/dL	3.9–86.4
C3c	19.5	mg/dL	90–180
C4	3.9	mg/dL	10–40
MPO-ANCA	Negative	U/mL	0–5
PR3-ANCA	Negative	U/mL	0–5
ANA	1:1,280		Negative
RF	2.4	IU/mL	0–15
ACPA	<8.00	U/mL	0–16
Anti-dsDNA IgG Ab	Positive: 39.4	IU/mL	0–20
Anti-Smith Ab	Positive: 200	U/mL	0–15
Anti-phospholipid Ab IgG	Positive: 19.2	GPL U/mL	0–10
Anti-cardiolipin Ab IgG	Positive: 17.2	GPL U/mL	0–10
Anti-β2 glycoprotein I IgG	Positive: 16.4	U/mL	0–5
Angiotensin-converting enzyme	30.1	U/L	13–63
Cryoglobulin	Negative		Negative

WBC, white blood cell; ESR, erythrocyte sedimentation rate; CRP, C-reactive protein; AST, aspartate aminotransferase; ALT, alanine aminotransferase; BUN, blood urea nitrogen; LDH, lactate dehydrogenase; A/G, albumin/globulin; IgG, immunoglobulin G; IgA, immunoglobulin A; IgM, immunoglobulin M; IgG4, immunoglobulin G4; C3c, complement component 3c; C4, complement component 4; MPO-ANCA, myeloperoxidase-specific antineutrophil cytoplasmic antibody; PR3-ANCA, proteinase 3-specific antineutrophil cytoplasmic antibody; ANA, antinuclear antibody; RF, rheumatoid factor; ACPA, anti-cyclic citrullinated peptide antibody; Anti-dsDNA, anti-double-stranded DNA; Ab, antibody; GPL, IgG phospholipid.

**Table 2.** 2019 European League Against Rheumatism/American College of Rheumatology Systemic lupus erythematosus classification score in the present case

Domain and criterion	Points
Clinical domain	
Fever	2
Leukopenia	3
Acute cutaneous lupus	6
Immunological domain	
Anti-phospholipid Ab positivity (positive anti-β2 glycoprotein I IgG)	2
Low C3 and low C4	4
Anti-dsDNA IgG or anti-Smith Ab	6
Total score	23

Antinuclear antibody positivity served as entry criterion and was not scored. IgG, immunoglobulin G; C3, complement component 3; C4, complement component 4; Anti-dsDNA, anti-double-stranded DNA; Ab, antibody.
